# Racial and ethnic disparities in pediatric firearm deaths persist in 2022 and 2023

**DOI:** 10.1186/s40621-025-00571-3

**Published:** 2025-03-24

**Authors:** Rafael Klein-Cloud, Bailey Roberts, Emma Cornell, Colleen Nofi, Chethan Sathya

**Affiliations:** 1 Division of Pediatric Surgery, Cohen Children’S Medical Center, Queens, NY USA; 2 Institute for Health System Science, Feinstein Institutes, Queens, NY USA; 3 Center for Gun Violence Prevention, Northwell Health, Queens, NY USA; 4 Donald and Barbara Zucker School of Medicine at Hofstra/Northwell, Hempstead, USA

**Keywords:** Firearm violence, Pediatric trauma, Racial disparities

## Abstract

**Background:**

Firearms became the leading cause of death in the United States pediatric population in 2019 and have persisted as the leading cause through 2021, with widening racial and ethnic disparities. We aimed to examine recent trends in U.S pediatric firearm mortality, how they differ by intent, and identify which ages, and racial and ethnic groups have been most impacted over time.

**Methods:**

The Centers for Disease Control and Prevention Wide-Ranging Online Data for Epidemiologic Research database was queried for mortalities in children aged 0–19 years from 2014–2023, and crude death rate was reported as number of deaths per 100,000 persons per year.

**Results:**

Firearms continued to be the leading cause of death in patients aged 0–19 years from 2021 to 2023, firearm crude death rate decreased from 5.8 to 5.5. In patients aged 14–19, firearms became the leading cause of death in 2016. In patients aged 0–13 years, firearms continue to be the fourth leading cause of death. Firearm death rates for Black children decreased from 18.6 in 2022 to 17.6 in 2023 yet remained far higher than other races, and highest in all census regions. Crude firearm death rates for American Indian and Alaskan Native (AIAN) children remained the second highest. The firearm suicide rate in Black children (1.8) surpassed that of White children (1.6) in 2022 and was the highest of any ethnicity in 2023. NonCore (rural) regions had the highest firearm crude death rates in 2018–19, and AIAN children were disproportionately affected in these areas, while Large Central Metro areas surpassed this in 2020–2021.

**Conclusions:**

Firearms remain the leading cause of death among children aged 14–19, and the fourth leading cause of death among children 13 and younger. Racial and ethnic disparities remain prominent, as Black and American Indian and Alaskan Native children continue to be disproportionately affected, particularly by firearm suicide. Prevention strategies should target these vulnerable populations and children at highest risk to prevent future firearm deaths.

## Background

In 2019 firearms became the leading cause of death among the pediatric population in the United States (US), surpassing motor vehicle traffic (or motor vehicle crashes) for the first time [1]. For many years, rates of firearm mortality in the pediatric population have been increasing. Previous increases in firearm mortality had been driven by increasing firearm suicides, with a decrease in firearm homicides between 2007 and 2014 [2]. In 2020, there was a substantial increase in the pediatric firearm death rate compared with smaller incremental increases over previous years, and this increase was largely driven by homicides [3]. Many speculated that this large increase was related to the 2019 coronavirus disease (COVID-19) pandemic as increases in social isolation, disruption to vital social services, and significant increases in firearm purchasing occurred during this time [4]. Along with the disproportionally large increase in firearm deaths in 2020, firearms continued to be the leading cause of death in the pediatric population in 2021, with notable racial disparities. Black children were disproportionately affected, with disparities worsening in 2021 even after the initial spike in 2020, again largely driven by homicides. Firearm homicides in Black children aged 0–19 years were 11 times higher than the rates in other races in 2021 [3]. Hispanic children and young adults have had higher rates of firearm mortality [5]. Historically, pediatric suicides by firearm have disproportionately affected White children [6–8]. In 2021 White children continued to be predominantly affected by firearm suicide, accounting for 78.4% of firearm suicides, although there was a significant increase in crude death rate by firearm suicide in Black children as well [3].

As pediatric firearm deaths occur less frequently compared to adults, this population presents challenges in studying the granularity of risk by age. Olufaje et al. (2020) used the National Trauma Data Bank to stratify the pediatric population into children (aged < 13 years) and teenagers (13–19 years), and found that intent significantly varied by age, with a higher percentage of unintentional firearm deaths in children, while teenagers had a higher percentage of firearm homicide deaths [7]. However, these categories are based on age and not on firearm mortality risk. Another study stratifying risk by age found that children aged 13–17 years had a 12-fold higher rate of fatal firearm injury than children younger than 13 [2]. The subdivision of the pediatric population based on risk of firearm mortality needs more examination.

Laws governing firearms are widely disparate across the country, varying by state [9]. In 2021, higher firearm death rates were increasing in southern and some western states [3]. The belief that firearm death is predominantly an urban issue is erroneous, with studies showing impact across the rural–urban continuum [10]. There is a paucity of data regarding pediatric firearm mortality analyzed by urbanization, and thus this study aims to elucidate these nuances and their relationship to racial disparities.

Since pediatric firearm deaths continued to increase into 2021 with widening racial disparities, this study aimed to determine whether this trend continued in 2022 and 2023, while further elucidating varying trends by age, race or ethnicity, and urbanization and geographic categories. Considering the significant effect that the COVID-19 pandemic had on firearm violence in the US [4], understanding trends post-pandemic can aid in developing a novel prevention approach. Prevention strategies and policies must be tailored to the specific population and type of violence, including targeted interventions that vary by intent (homicide, suicide, unintentional) [9, 11]. Understanding the aspects of firearm violence that predominantly contribute to these trends has implications for violence interruption and suicide prevention at a community level along with state and federal legislation and policy.

## Methods

We utilized the Centers for Disease Control and Prevention (CDC) Wide-ranging Online Data for Epidemiologic Research (WONDER) data query system. This data set uses mortality data from death certificates in all 50 states and Washington, District of Columbia, and includes underlying causes of death and demographics. The CDC WONDER system was queried for firearm mortality data, and other leading traumatic causes of death in children and adolescents aged 0–19 years from 2014–2023 [12, 13]. To remain consistent with pediatric literature, we used 0–19 years to define the pediatric age group [7]. The underlying cause of death was narrowed by International Classification of Diseases 10th Revision codes (ICD-10) for injury intent (homicide, suicide, unintentional, undetermined, legal intervention/operations of war) and mechanism (firearm, motor vehicle traffic, poison, suffocation, etc.). Further stratifications of this data were performed for age, race, ethnicity, urbanization, and census region [12, 13]. The 2013 National Center for Health Statistics Urban–Rural Classification Scheme for Counties was used to define urbanization [14]. The United States census regions used were Northeast, Midwest, South, and West.

The CDC WONDER database reports absolute mortality numbers, except statistics representing 1–9 deaths, which are suppressed to protect the privacy of the individual(s). Crude death rates are reported, except where absolute values of deaths < 20 make this calculation unreliable. Crude death rate is defined as the number of deaths reported each calendar year per 100,000 persons [12, 13].

## Results

### Leading causes of death

**Fig. 1:**
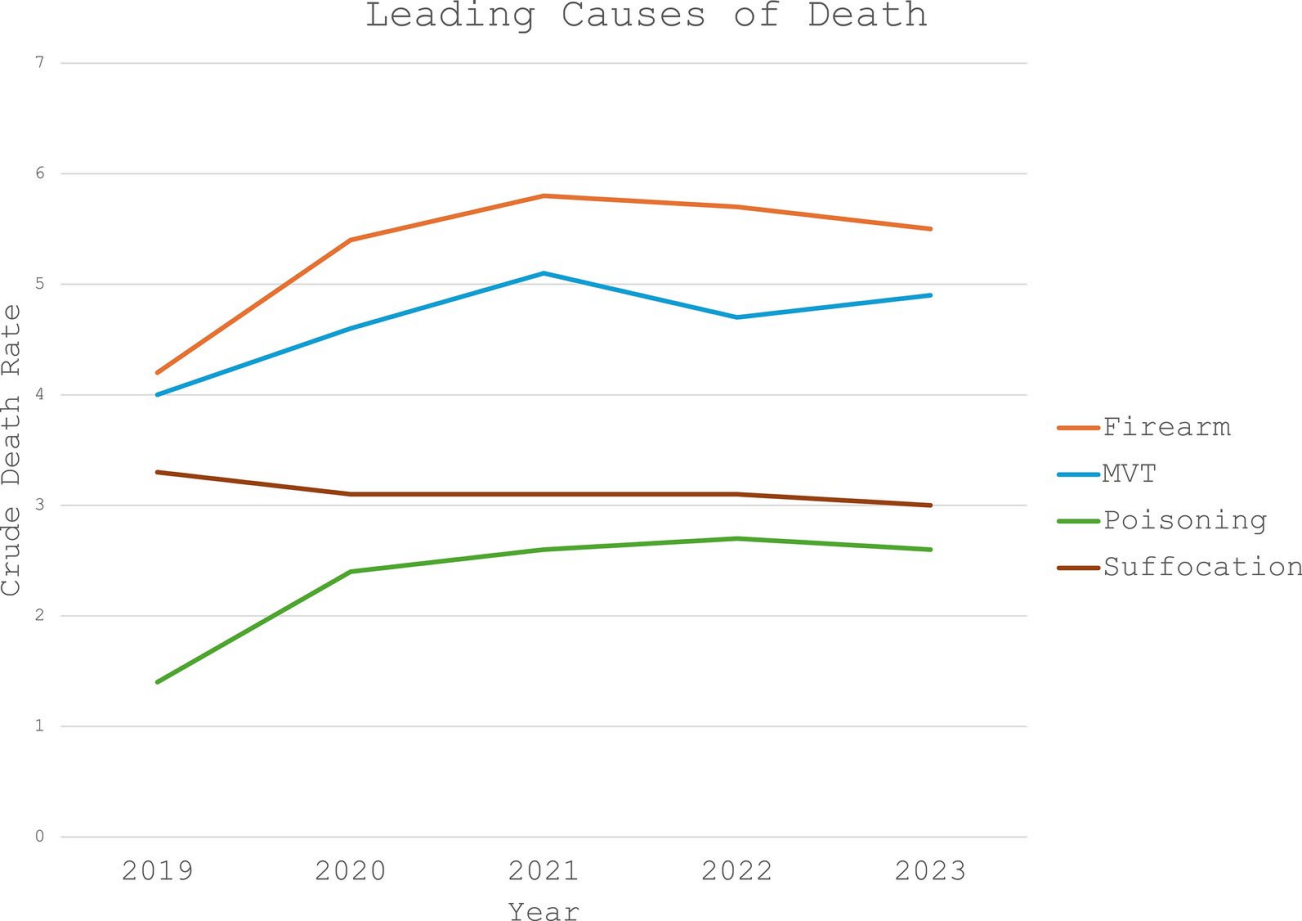
Leading causes of traumatic death. Crude death rate for patients 0–19 years of age stratified by mechanism of injury. Crude death rate represents deaths per 100,000 population. MVT = Motor Vehicle Traffic

In 2023 the crude death rate due to traumatic injury and poisoning in patients 0–19 years of age was 19.6 per 100,000 population, down from 20.0 in 2022 and 20.3 in 2021, a 3.5% decrease since the 5-year peak in 2021. Similarly, the overall rate of firearm deaths decreased from 5.8 per 100,000 population in 2021 to 5.7 in 2022, and to 5.5 in 2023, with the 2023 crude death rate representing a 5.2% decrease from the 2021 peak. Firearms continued to be the leading cause of death in children aged 0–19 (Fig. 1). The second leading cause of death continued to be motor vehicle traffic (MVT), with a crude death rate of 4.7 per 100,000 population in 2022 and 4.9 in 2023. The next two leading causes of death were suffocation followed by poisoning, both of which also decreased in 2023 from 2022.

**Fig. 2:**
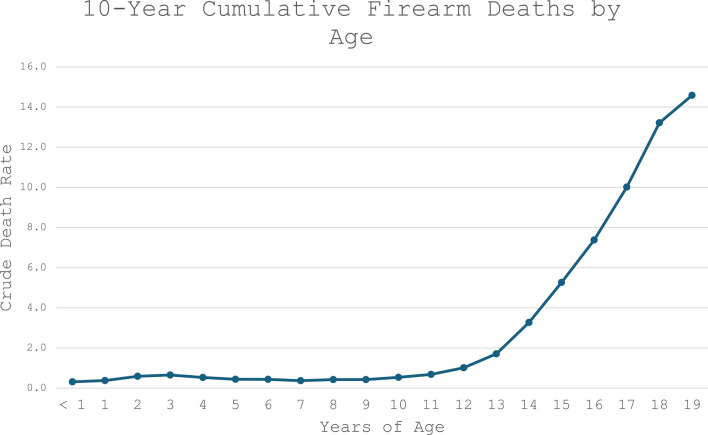
10-Year cumulative firearm deaths by age. Crude death rates were calculated for each year of age for firearm deaths from 2014–2023. Crude death rate represents deaths per 100,000 population

To stratify by age, the cumulative number of firearm deaths over 10 years from 2014–2023 were calculated for each year of age, showing a sharp increase in number of firearm deaths starting at age 13, suggesting that at 14 years children start to become higher risk for firearm death (Fig. 2). Therefore, the pediatric population was stratified into patients less than 14 years, and 14–19 years of age.

**Fig. 3:**
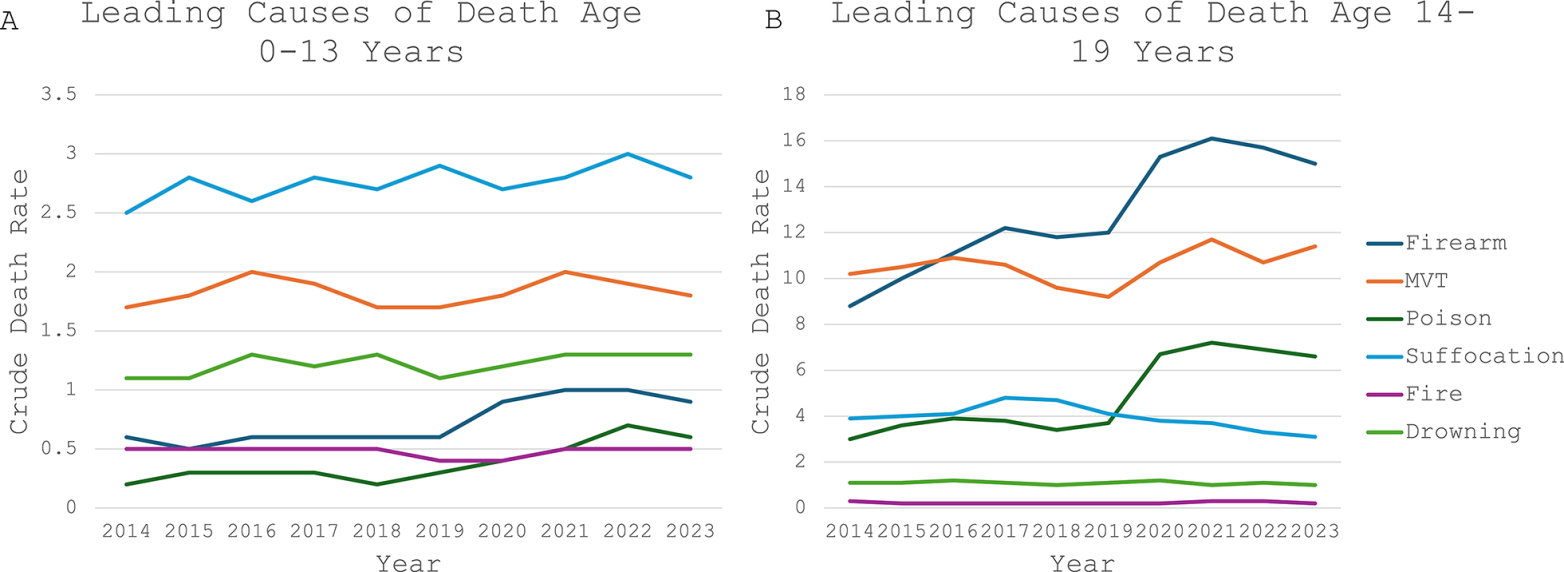
Leading causes of death stratified by age. Crude death rate represents deaths per 100,000 population. **A **Ages 0–13 years. **B **Ages 14–19 years

The leading traumatic causes of death in children less than 14 years of age in 2023 were suffocation (crude death rate of 2.8 per 100,000 population), followed by MVT (crude death rate of 1.8), and drowning (crude death rate of 1.3). Firearms were the fourth leading cause of death with a crude death rate of 1.9 per 100,000 population. In 2020, firearm mortality rates increased by 50% compared to 2019, and crude death rate has remained relatively stable (Fig. 3A).

The leading traumatic causes of death in children aged 14–19 in 2023 were firearms (crude death rate of 15 per 100,000 population), followed by MVT (crude death rate of 11.4), then poisoning (crude death rate of 6.6). In children aged 14–19, firearms became the leading cause of death in 2016 with a crude death rate of 11.1 per 100,000 population, surpassing MVT with a crude death rate of 10.9 (Fig. 3B).

### Firearm deaths by intent

**Fig. 4:**
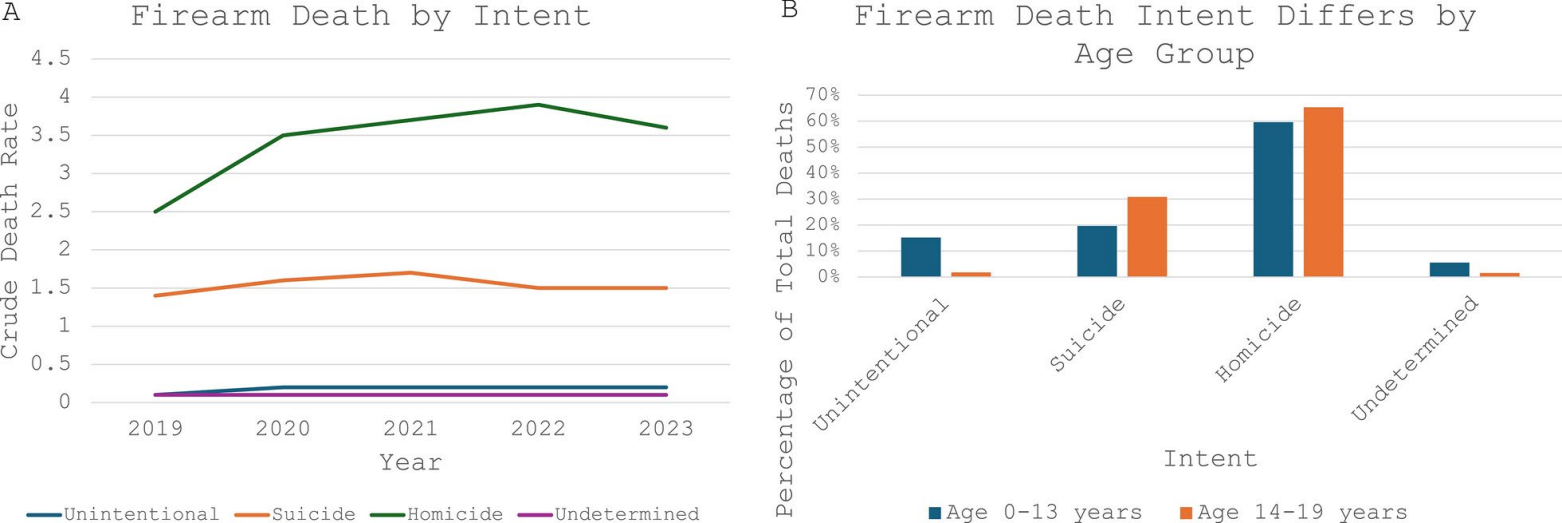
Firearm deaths by intent. **A **Firearm deaths by intent for children aged 0–19 years. Mortalities due to legal intervention or operations of war in are too low for the CDC WONDER database to calculate a crude mortality rate, so that data is excluded from this figure. Crude death rate represents deaths per 100,000 population. **B **Firearm deaths by intent from 2019–2023, comparing children aged 0–13 years to children aged 14–19 years

In 2023, for pediatric patients aged 0–19 years, the firearm homicide rate decreased to 3.6 per 100,000 population, down 7.7% from 3.9 in 2022, yet up from 3.7 in 2021. The firearm suicide rate began to decrease, from 1.7 per 100,000 population in 2021 to 1.5 in 2022, a 12% decrease, and remaining at 1.5 in 2023. The crude death rates due to unintentional and undetermined intents remained low and stable (Fig. 4A). The numbers for death by legal intervention or operations of war in this population were too low to calculate a reliable crude death rate, but the absolute numbers have increased from 13 deaths in 2021, 19 deaths in 2022, to 29 in 2023.

Homicide was the most common intent among firearm deaths even when stratifying by age. Over the five-year period from 2019–2023, homicide accounted for 59.6% of all firearm deaths in children 0–13 years, and 65.3% of deaths among youth ages 14–19 years. The percentage of firearm deaths due to suicide was greater in youth aged 14–19 years, with 30.8% in children under aged 14–19 years and 19.7% in children ages 0–13 years. A majority (92.5%) of all firearm suicides occurred in children aged 14–19. Children 0–13 years accounted for a greater percentage of unintentional firearm deaths (15.2%) compared to children ages 14–19 (1.8%) (Fig. 4B). In 2023, the crude death rate for unintentional firearm injuries in Black children was 0.5 per 100,000 persons, fivefold higher than that of White children, at 0.1, and this trend remained consistent since 2019.

### Racial and ethnic disparities

**Fig. 5:**
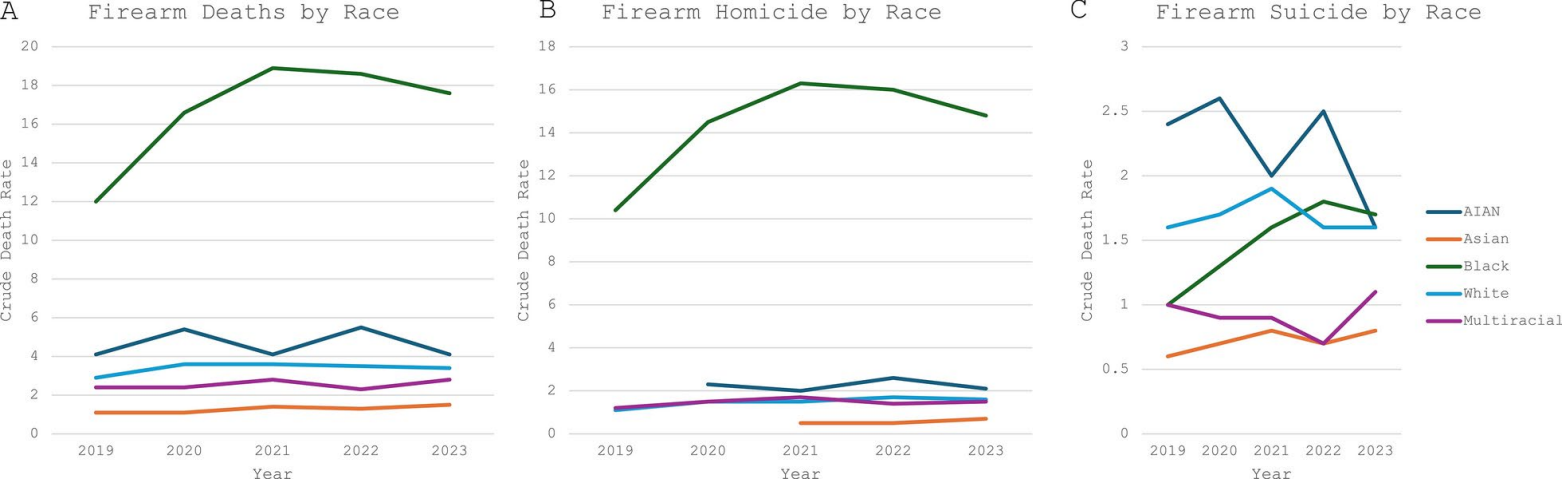
Trends in Crude Firearm Death Rate by Race, age 0–19. Crude death rate represents deaths per 100,000 population. AIAN = American Indian Alaskan Native. **A **Firearm deaths by race. **B **Firearm homicide deaths by race. **C **Firearm suicide deaths by race

Substantial racial disparities among pediatric firearm deaths persisted in 2022 and 2023. Black children continued to be the most disproportionately affected, with a firearm death rate of 18.6 per 100,000 population in 2022, decreasing by 5.4% in 2023 to 17.6, compared to White children with a firearm death rate of 3.5 in 2022 and 3.4 in 2023. The next highest firearm death rate occurred among American Indian and Alaskan Native (AIAN) children, with a firearm death rate of 5.5 per 100,000 population in 2022 and 4.1 in 2023—one of the few groups among which firearm death rates increased in 2022 but decreased in 2023 (Fig. 5A).

Among AIAN children, there was a 30% increase in the firearm homicide rate from 2.0 per 100,000 population in 2021 to 2.6 in 2022. There was a 13.3% increase in firearm homicides in 2022 among White children, with a rate of 1.7 per 100,000 population in 2022, increasing from 1.5 in 2021. In 2023, firearm homicide rates increased in Asian and multi-racial children, and decreased in AIAN, White, and Black children. While the firearm homicide rate in Black children decreased 7.5% from 16 per 100,000 population in 2022 to 14.8 in 2023, this rate was still sevenfold higher than the next highest of 2.1 in the AIAN population (Fig. 5B).

In 2022, the firearm suicide rate in Black children was 1.8 per 100,000 population, which surpassed that of White children for the first time, whose firearm suicide death rate was 1.6. In 2023, for the first time, Black children had the highest firearm suicide crude death rate of any race with 1.7 per 100,000 population, as the rate of firearm suicide in AIAN children fell by 36% from 2.5 in 2022 to 1.6 in 2023. From 2019–2022, firearm suicide rates were highest among AIAN children (Fig. 5C).

Firearm mortality among Hispanic children increased 10% from 4.0 per 100,000 population in 2021 to 4.4 in 2022 and then decreased to 4.3 in 2023. There was a corresponding decrease in firearm death rate in non-Hispanic individuals from 6.4 per 100,000 population in 2021 to 6.2 in 2022, and 5.9 in 2023. When assessing drivers of the increase in 2022, the firearm homicide rate in the Hispanic population increased from 2.8 per 100,000 population in 2021 to 3.3 in 2022, decreasing to 3.2 in 2023. The firearm suicide rate in the Hispanic population decreased from 1.0 per 100,000 population in 2021, to 0.8 in 2022, and back up to 0.9 in 2023. Analyzing 5-year data from 2019–2023, the overall firearm crude death rate of Hispanics was 3.9 per 100,000 persons, that of non-White Hispanics was 8.5, and that of White non-Hispanics was 3.1.

### Urbanization and geography

**Fig. 6:**
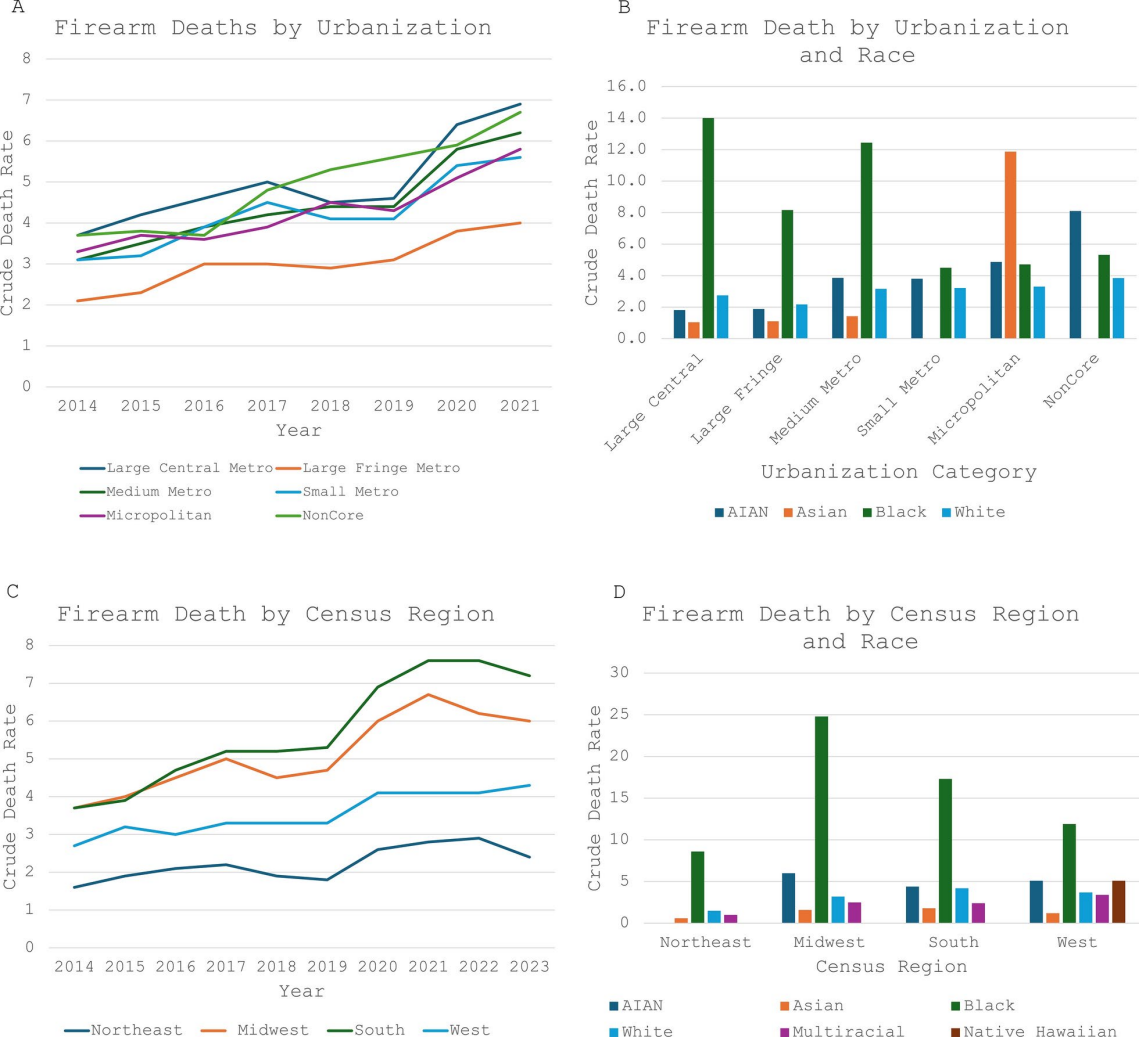
Firearm Deaths by Urbanization. Crude death rates are per 100,000 population. AIAN = American Indian and Alaskan Native. **A** Firearm Deaths by Urbanization. **B** Firearm Deaths by Urbanization and Race: Data for Asian children in Small Metro and Micropolitan categories are suppressed due to low numbers. **C** Firearm Death by Census Region. **D** Firearm Death by Census Region and Race: Data for AIAN children in Northeast region are suppressed due to low numbers. Data for Hawaiian Native children are suppressed due to low numbers in all regions except West

Data regarding urbanization were not available from CDC WONDER for 2022 and 2023, so firearm deaths from 2014–2021 were analyzed. NonCore (rural) regions had the highest firearm crude mortality rate in 2018 and 2019 (5.3 and 5.6 per 100,000 persons, respectively). From 2019 to 2020 firearm crude death rate increased 39% in Large Central Metro regions. Thus Large Central Metro regions achieved the highest firearm crude death rate compared to other urbanization categories in 2020 and 2021 (6.4 and 6.9 per 100,000 persons, respectively). Analyzing the racial breakdown by urbanization, Black children were disproportionately affected in all region types except micropolitan and NonCore regions. In micropolitan regions, Asian children had a crude firearm death rate 2.4 times greater than the next highest of AIAN children, and Black children in this region had the third-highest firearm crude death rate. In NonCore regions, AIAN children had the highest firearm crude death rate (Fig. 6 A and B).

For the last 10 years, the South census region has had the highest firearm crude death rate, except for 2015, when the Midwest had a higher firearm crude death rate. Looking at the racial breakdown by census region, Black children were disproportionately affected in all regions. In the Midwest region Black children had a firearm crude death rate 4 times higher than AIAN children, who had the next highest crude death rate. In the Northeast region, Black children were affected 5.7 times more than White children, the race with the next highest crude death rate. AIAN children were the second-most impacted in all regions except the Northeast (Fig. 6 C and D).

## Discussion

Firearms continue to be the leading cause of mortality in US children 0–19 years; however, rates are decreasing marginally. The lower overall firearm mortality contributes to the overall decrease in crude death rate due to all traumatic injuries in this population, however this decrease is offset by an increase in deaths due to motor vehicle traffic injuries. Despite this decrease, in 2023 4,470 children were killed by firearms, while 3967 children were killed by motor vehicle crashes. The scale of pediatric firearm deaths represents a uniquely American epidemic [15]. In 2010, among high-income countries around the world, 91% of firearm deaths in children occurred in the US [16], and this percentage increased to 98.1% in 2015 [17].

While it is widely cited that firearms became the leading cause of death in children 19 years and younger in 2019 [1], this change occurred in 2016 for adolescents aged 14–19, indicating that this epidemic has been ongoing for longer than previously thought. Among the younger age group of children less than 14, firearms remain the fourth-leading cause of death, and in this population, there is a higher percentage of unintentional firearm death. The variation of firearm death rate and different intents with age demonstrates the need for targeted interventions for specific populations. For example, the younger population may benefit more from safe firearm storage counseling and education [18], while the older population of children may benefit from safe storage education [18], behavioral health resource utilization [19], and hospital-based violence intervention programs (HVIPs) [20].

Racial disparities persist in pediatric firearm mortality, as Black and AIAN children continue to be disproportionately affected in recent years. Race and ethnicity in the United States are highly correlated with poverty levels [21]. In 2021, 10% of non-Hispanic White children and Asian children experienced poverty, whereas 23% of Hispanic children, 30% of AIAN children, and 31% of non-Hispanic Black children experienced poverty [22]. All-cause mortality, including firearm mortality in pediatrics, correlate with higher poverty levels and lower socioeconomic status, even when controlling for demographic variables [22]. Additionally, prior evidence has found pediatric firearm mortality rates are historically higher in communities of color [7, 23]. While this study did not address the correlation of poverty levels to the recent changes in firearm death rates, the strong ties in the US between race and poverty and the heterogeneity of situations surrounding firearm deaths reflect a mixture of disparities including poverty, neighborhood violence, food and healthcare access, government structure, and other social determinants of health contributing to increased firearm mortality [3, 9, 18]. Dismantling the root causes of these different factors is one step to addressing firearm mortality disparities among Black, American Indian and Alaska Native, and Hispanic children.

Firearm mortality disparities in Black children have largely been driven by homicides, but firearm suicides among Black children have been increasing steadily over the past 5 years, with a 91% increase between 2019 and 2022. For the first time, the firearm suicide rate in Black children surpassed that of White children in 2022. In 2023, there was a slight decrease in firearm suicide mortality in Black children, and despite this, the firearm suicide rate of Black children was highest of any race, surpassing AIAN children for the first time. Historically, White children have been disproportionately affected by firearm suicides and still comprise most suicides by absolute numbers [24]. Black children are now disproportionately affected by both firearm homicides and suicides. This striking new trend highlights the need to focus on this vulnerable population and increase targeted interventions that address suicidality, mental health resources, and root causes of impulsive suicidal behavior, in addition to the need for focus on prevention of homicides with community engagement, violence intervention programs and limiting firearm access [19, 25–27]. Interventions may include education for families, screening for depression and suicidal ideation, and lethal means counseling for children and adolescents, while also addressing root causes of violence and depression which are highly correlated with environment, socioeconomic status, education and poverty.

American Indian and Alaskan Native (AIAN) children remain highly vulnerable to firearm mortality. Despite this alarming trend, there is a paucity of research regarding firearm violence in this population [23]. Recognizing the unique qualities among this population, including differences in tribal political and legal structure is important to identify protective factors and design interventions that support this unique demographic [28]. According to the CDC, AIAN individuals comprised 1.5% of the US population in 2020 [28]. Despite the relatively small population, the AIAN population has been noted to have high rates of firearm suicide [2, 3]. With the firearm mortality rates persistently high, there is dire need to understand the underlying drivers behind this trend. Intimate partner violence has been shown to be a major driver of homicide in this population [29]. Further, in a 2023 survey, AIAN respondents reported high firearm access rates, predominantly handguns owned for home protection, citing need for self-protection and a lack of faith in police. Notably, 37.2% reported always storing at least one firearm loaded [30]. While firearm ownership is common in AIAN communities, simultaneously there has been historic disinvestment into tribal and community support services creating significant barriers to adequately addressing these disparities. Little research has been done into drivers of pediatric-related firearm violence in this population, which may drastically differ from those seen in adults. Prevention and intervention strategies must recognize the unique cultural, political and social circumstances among AIAN communities and their children to appropriately address existing disparities.

Hispanic children had lower firearm mortality rates compared to non-Hispanic children, and this held true for both homicides and suicides. A recent study showed that in patients ages 5–84 years, the firearm death rate among Hispanic patients was greater than that of White non-Hispanics [5], and this study confirms continuation of that trend. The Hispanic population is the largest minority group in the US [5], encompassing multiple races, hence why it is important to understand the nuances of how firearm injuries affect this population. Non-White Hispanics had the highest firearm crude mortality rate from 2019–2023, compared to White Hispanics, and White non-Hispanics, indicating that this population would benefit from a focus on firearm homicide and suicide prevention.

This study further confirms that pediatric firearm death is not solely an urban issue, with highest rates oscillating between Large Central Metros and NonCore (rural) regions. This study further analyzed the racial breakdown across urbanization category and census region, and found that Black children are disproportionately killed by firearms in all urbanization categories except Micropolitan and NonCore, and in all census regions. Asian and AIAN children are most affected in Micropolitan regions, and AIAN children in NonCore regions, further highlighting AIAN children as a vulnerable population. While there is variable efficacy of various types of firearm laws [9], research shows that areas with stricter firearm legislation have lower rates of firearm violence [9, 11, 31, 32] Identifying high risk regions, at municipal to federal levels, is important for identifying which of these laws may be effective for the population under its jurisdiction.

This study has several limitations. It is a retrospective study of a large database, and despite this, the number of pediatric firearm mortalities in certain areas is small, requiring CDC WONDER database to report crude death rate as “unreliable” [12, 13]. Previous work has demonstrated that ICD-10 codes for firearm injury do not always accurately describe the true intent of the firearm injury [33]. Furthermore, the CDC WONDER database does not capture non-fatal firearm injuries, and thus the larger scope firearms morbidity was not analyzed in this study. This is particularly important when comparing intent of violence, as mortality from suicide by firearm is far higher than that of other intents [34, 35]. The urbanization CDC WONDER data are only available through 2021, so additional work must be done to identify more recent trends. Further studies addressing total burden and disparities of non-fatal firearm injuries in children in the US are necessary to continue to inform interventions, laws, and policies that may prevent future injury and death.

## Conclusions

Death due to firearms became the leading cause of death of children aged 14–19 years in 2016 and continues to be the leading cause of death in the overall pediatric population, but not in children aged 0–13 years. Despite overall decreases in mortality, both from homicides and suicides, disparities remain prominent. Black children are disproportionately affected with significantly higher rates of firearm homicides than all other races, and for the first time reaching the highest rate of firearm suicide of all races in 2023. AIAN children are also significantly affected, until 2023 having the highest rates of suicide compared to any other race and persistently second highest overall rate of firearm death. Targeted interventions that dismantle the root causes of suicide and homicide in these populations are necessary to prevent future firearm injuries and address these disparities.

## Data Availability

The datasets analyzed during the current study are available in the Center for Disease Control and Prevention Wide-ranging Online Data for Epidemiologic Research (CDC WONDER) database: https://wonder.cdc.gov/.

## Abbreviations

US: United States
COVID-19: Coronavirus disease of 2019
CDC: Centers for Disease Control and Prevention
WONDER: Wide-ranging Online Data for Epidemiologic Research
ICD-10: International Classification of Diseases 10th Revision
MVT: Motor vehicle traffic
AIAN: American Indian and Alaskan Native
